# Multifunctional Nanomaterials for Advancing Neural Interfaces: Recording, Stimulation, and Beyond

**DOI:** 10.1021/acs.accounts.4c00138

**Published:** 2024-06-11

**Authors:** Daniel Ranke, Inkyu Lee, Samuel A. Gershanok, Seonghan Jo, Emily Trotto, Yingqiao Wang, Gaurav Balakrishnan, Tzahi Cohen-Karni

**Affiliations:** † Department of Materials Science and Engineering, Carnegie Mellon University, Pittsburgh, Pennsylvania 15213, United States of America; ‡ Department of Biomedical Engineering, Carnegie Mellon University, Pittsburgh, Pennsylvania 15213, United States of America

## Abstract

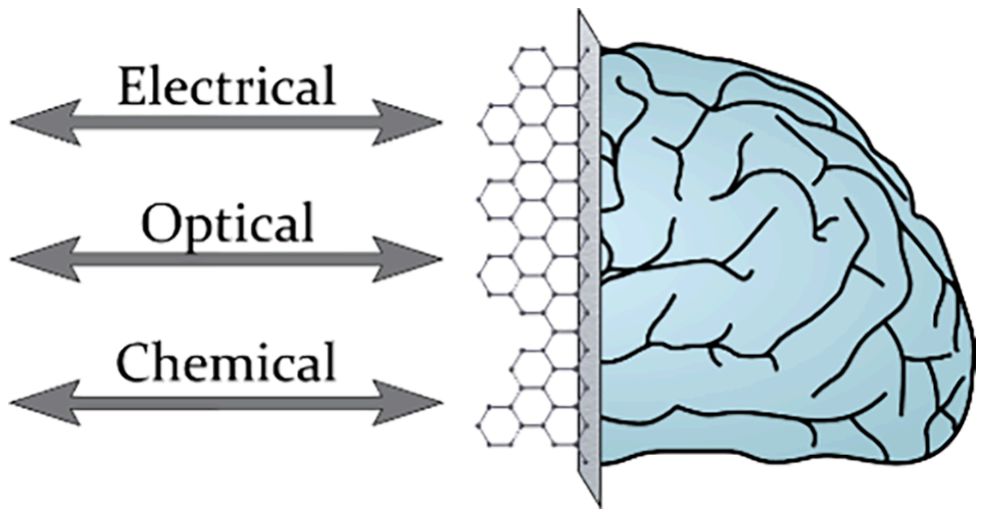

Neurotechnology has seen dramatic improvements in the last three decades. The major focus in the field has been to design electrical communication platforms with high spatial resolution, stability, and translatability for understanding and affecting neural pathways. The deployment of nanomaterials in bioelectronics has enhanced the capabilities of conventional approaches employing microelectrode arrays (MEAs) for electrical interfaces, allowing the construction of miniaturized, high-performance neuroelectronics (Garg, R.; et al. *ACS Appl. Nano Mater.***2023**, *6*, 8495). While these advancements in the electrical neuronal interface have revolutionized neurotechnology both in scale and breadth, an in-depth understanding of neurons’ interactions is challenging due to the complexity of the environments where the cells and tissues are laid. The activity of large, three-dimensional neuronal systems has proven difficult to accurately monitor and modulate, and chemical cell–cell communication is often completely neglected. Recent breakthroughs in nanotechnology have provided opportunities to use new nonelectric modes of communication with neurons and to significantly enhance electrical signal interface capabilities. The enhanced electrochemical activity and optical activity of nanomaterials owing to their nonbulk electronic properties and surface nanostructuring have seen extensive utilization. Nanomaterials’ enhanced optical activity enables remote neural state modulation, whereas the defect-rich surfaces provide an enormous number of available electrocatalytic sites for neurochemical detection and electrochemical modulation of cell microenvironments through Faradaic processes. Such unique properties can allow multimodal neural interrogation toward generating closed-loop interfaces with access to more complete neural state descriptors. In this Account, we will review recent advances and our efforts spearheaded toward utilizing nanostructured electrodes for enhanced bidirectional interfaces with neurons, the application of unique hybrid nanomaterials for remote nongenetic optical stimulation of neurons, tunable nanomaterials for highly sensitive and selective neurotransmitter detection, and the utilization of nanomaterials as electrocatalysts toward electrochemically modulating cellular activity. We highlight applications of these technologies across cell types through nanomaterial engineering with a focus on multifunctional graphene nanostructures applied though several modes of neural modulation but also an exploration of broad material classes for maximizing the potency of closed-loop bioelectronics.

## Key References

- GargR.; BalakrishnanG.; RashidR. B.; GershanokS. A.; RomanD. S.; WangY.; KouassiP. C.; RivnayJ.; Cohen-KarniT.Graphene and poly(3,4-ethylenedioxythiophene)–polystyrene sulfonate hybrid nanostructures for input/output bioelectronics. ACS Appl. Nano Mater.2023, 6( (10), ), 8495–850510.1021/acsanm.3c00849.^1^*The heterostructure of graphene/PEDOT:PSS as electrodes exhibited a greater than 2 orders of magnitude in charge injection capacity increase and lower impedance than planar metal electrodes, posing as a high potential platform for bidirectional neural interfaces.*
- WangY.; GargR.; HartungJ. E.; GoadA.; PatelD. A.; VitaleF.; GoldM. S.; GogotsiY.; Cohen-KarniT.Ti3C2Tx MXene flakes for optical control of neuronal electrical activity. ACS Nano2021, 15( (9), ), 14662–1467110.1021/acsnano.1c0443134431659 PMC9285622.^2^*Titanium carbide MXene nanosheets enabled the photothermal stimulation of neurons with light intensity comparable to that of optogenetics and high spatiotemporal resolution.*
- CastagnolaE.; GargR.; RastogiS. K.; Cohen-KarniT.; CuiX. T.3D fuzzy graphene microelectrode array for dopamine sensing at sub-cellular spatial resolution. Biosen. Bioelectron.2021, 191, 11344010.1016/j.bios.2021.113440PMC837678634171734.^3^*The graphene nanostructure was applied as a microelectrode for the detection dopamine with sensitivities in the nM range, selectivity against serotonin, and an electrode area of 2 μm*^2^*with fast scan cyclic voltammetry.*
- San RomanD.; KrishnamurthyD.; GargR.; HafizH.; LamparskiM.; NuhferN. T.; MeunierV.; ViswanathanV.; Cohen-KarniT.Engineering three-dimensional (3D) out-of-plane graphene edge sites for highly selective two-electron oxygen reduction electrocatalysis. ACS Catal.2020, 10( (3), ), 1993–200810.1021/acscatal.9b03919.^4^*Three-dimensional graphene nanostructures were optimized toward highly selective H*_2_*O*_2_*production via the oxygen reduction reaction in the 2-electron pathway.*

## Introduction

Neuronal signal transduction represents the basis for nearly every high-order process in the human body from cognitive function to neurological disease progression.^5^ As such, the capability to read and write into these signal pathways represents one of the greatest entryways to understanding and modulating health as well as our interaction with the world. Technology to enable neuronal interfaces has grown alongside the development in electronics, with the first example of modern neuronal stimulation in 1936^6^ and action potential recording in 1939^7^ leading up to modern systems consisting of thousands of channels and near immunological invisibility.^8,9^ These technological developments have led to more foundational neuronal function discoveries and versatile techniques for manipulation and recording than have historically been possible.

**Figure 1:**
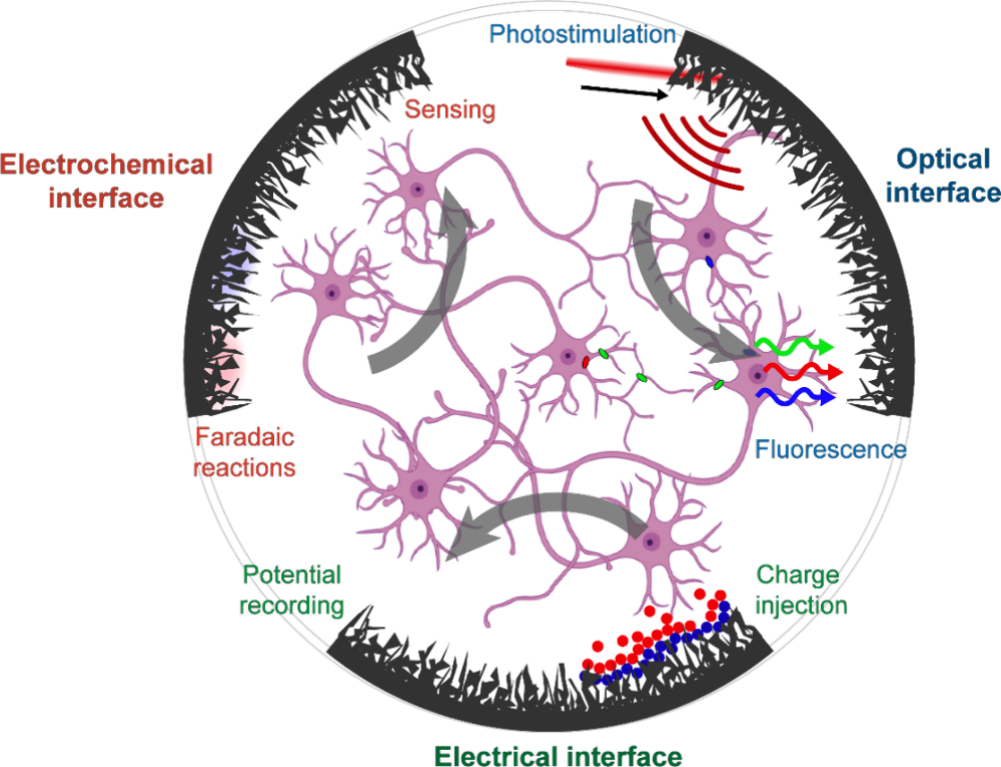
Nanomaterial-enabled bidirectional neuronal interfaces with electrochemical, optical, and electrical modalities. Electrochemical modulation is highlighted through the Faradaic neuron microenvironment control through selective catalysts with simultaneous biosensing, optical through laser-induced photostimulation of neural activity and multichannel fluorescent measurements, and electrical through input/output neural modulation and recording.

The neuronal action potential is a fundamental transient event marked by cell membrane potential changes due to the opening and closing of ion channels and pumps. The potential shift from peak initiation to stabilization at the baseline typically lies within subms to 20 ms for humans and spans approximately 100 mV from the membrane resting potential to peak potential.^5^ The membrane potential and intracellular/extracellular ion concentrations dictate the state of a neuron and form the basis of nearly all modern neuronal interfaces through measurement and manipulation. Numerous measurement and modulation techniques have been utilized, including electrical, optical, magnetic, acoustic, and electrochemical methods.^10−12^ The evolving demands of neural interfaces with minimal immunological response, single-unit spatiotemporal resolution, bidirectional communication, and clinical translation underscore the growing importance of the materials used in such applications. With the earliest forms of interfaces, planar-style titanium, platinum/iridium, and carbon electrodes were deployed for electrically mapping and modulating neural activity.^5^ Their limited performances (e.g., inferior signal-to-noise ratios (SNR) and high potential requirement for sufficient current injection^13^) impeded interfacing materials to the neuron effectively. The two most well-developed alternate modalities, optical and electrochemical, are also challenging for bidirectional neural interfaces for similar material-constrained limitations. For example, optical modulation of neurons requires optically active materials with high absorption in the near-infrared (NIR) window due to reduced biological absorption allowing greater penetration depths in tissues,^14^ while species-selective reactivity is required for electrochemical methods.^15^ With traditional material macro-engineering, such characteristic tunability is exceedingly difficult; however, materials engineered at the nanoscale have shown a considerable range in properties even across identical material classes (Figure 1).^1,4,15^

A nanoengineered material is a broad class consisting of any whose structure has been manipulated on an atomic to nanometer scale to manifest a particular characteristic such as electrical conductivity, surface nanoporosity, optical activity, or catalytic behavior. The discovery of nanocarbons is one of the most impactful outcomes in nanoscience, including graphene, carbon nanotubes (CNTs), nanodiamonds, and recently reported vertically oriented fuzzy graphene nanostructures.^16^ Other lower-dimensional materials, such as single-layer metal carbides (e.g., MXene) and metal/semiconductor nanowires, have been identified as both high-conductivity electrodes in bioelectronics and among the highest-performing optical neural stimulators.^4,10^ For direct measurements of the often subnanomolar chemical species involved in neural communication, nanostructured surfaces pose both abundant and tunable electrochemical sites critical for sensor selectivity/sensitivity.^4^ Recent developments in nanomaterial engineering have led to a surge in multimodal modulation and detection of neuronal activity.^17^ Nanomaterials’ advantages and further improvements will further open opportunities for unprecedented control and understanding of neural behavior through electrical, optical, and electrochemical techniques.

## Electrical Modulation and Recording

**Figure 2:**
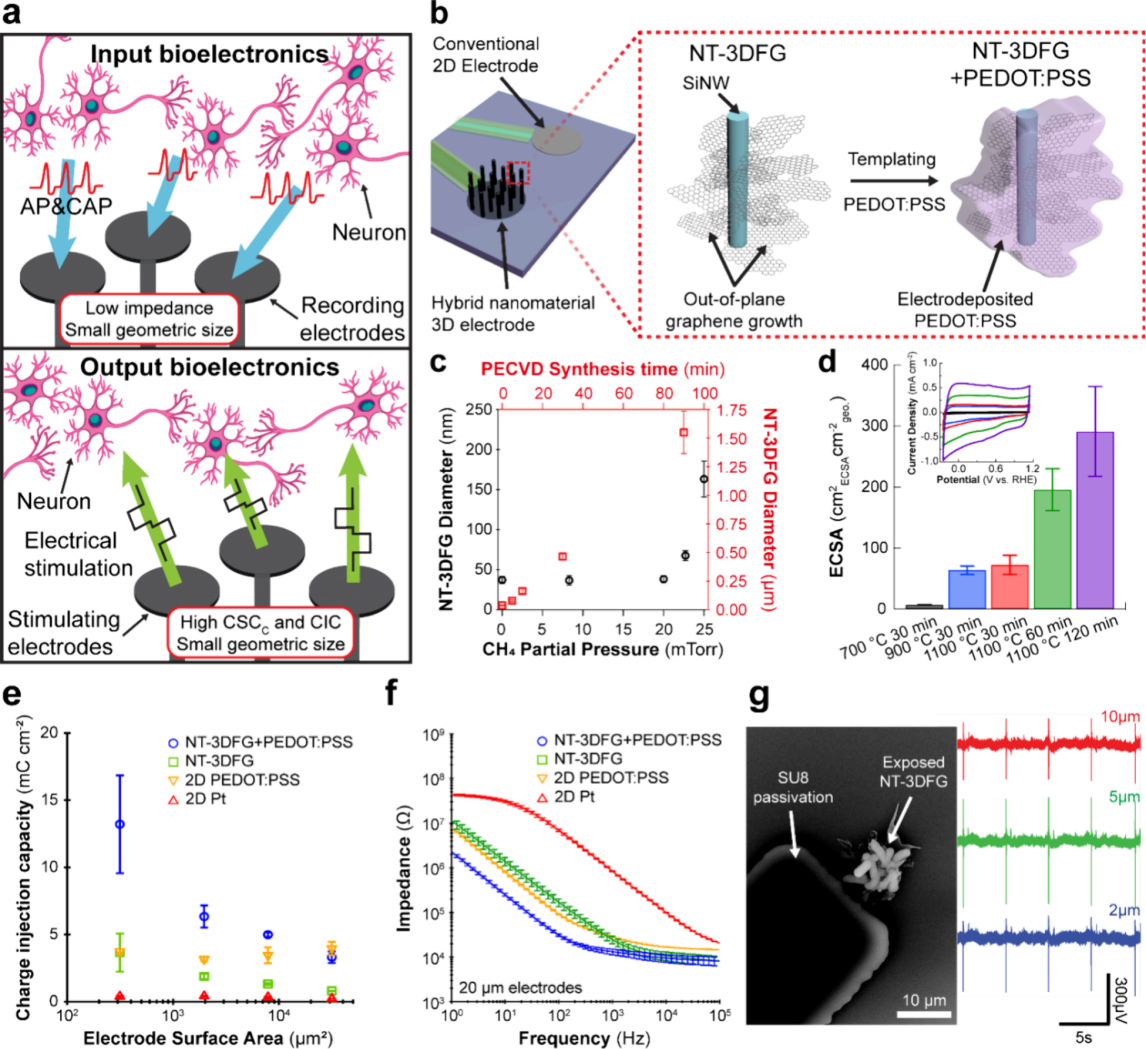
Multidimensional nanostructured hybrid materials for electrodes in input/output (I/O) bioelectronics. (a) Schematic illustrations of the operation of input and output bioelectronics; AP: action potential; CAP: compound action potential. (b) Schematic illustration of a conventional 2D and multidimensional nanostructured NT-3DFG electrode and synthesis of a hierarchical hybrid nanomaterial by conjugating PEDOT:PSS on NT-3DFG; SiNW: silicon nanowire. (c) Process parameter-driven NT-3DFG geometry tunability. (d) Electrochemical surface area (ECSA) for NT-3DFG; inset: the differential double-layer capacitance measurements for ECSA determination. (e) Charge injection capacity (CIC) characterization of Pt (red), PEDOT:PSS (yellow), NT-3DFG (green), and PEDOT:PSS-conjugated NT-3DFG (NT-3DFG+PEDOT:PSS, blue) microelectrodes as a function of the geometrical surface area. (f) 3D topography reduces electrode impedance. Impedance as a function of frequency for Pt (red), PEDOT:PSS (yellow), NT-3DFG (green), and NT-3DFG+PEDOT:PSS (blue). (g) High signal-to-noise ratio electrical activity of human embryonic stem cell derived cardiomyocytes (hESC-CMs) recorded via 2, 5, and 10 μm NT-3DFG microelectrodes. Figure reprinted with permission from ref (1), copyright 2023 American Chemical Society; ref (4), copyright 2020 American Chemical Society; ref (36), copyright 2017 American Chemical Society; and ref (37), copyright 2020 Springer Nature.

Input/output (I/O) bioelectronics enable real-time sensing (output) and stimulation (input) of cellular and tissue activity (e.g., electrophysiology) through direct electrical charge injection and signal acquisition.^18^ They represent the forefront of neural interface clinical translation with realized applications in neurological disease diagnosis and therapeutic (e.g., epilepsy detection and deep brain stimulation for tremor control, respectively).^19,20^ Sensing and stimulation of electrophysiological activity through I/O bioelectronics rely on the spatiotemporal distribution of charges at the electrode-cell/tissue interface.^21^ Input bioelectronics induce local changes in electrochemical potentials by injecting charge at the interface, while output bioelectronics detect local changes in the cellular membrane potential induced by the generation and propagation of single and compound action potentials (Figure 2a).^5,22^ Therefore, the performance of I/O bioelectronics relies on the functional properties of the constituent electrode materials. Key factors include low electrochemical impedance for electrophysiological recordings and efficient charge injection for stimulation.^23,24^ Additionally, the interface between electrode materials and biological systems plays a crucial role.^16,25−27^ Traditional bioelectronic electrodes are two-dimensional (2D) and rely on metals (e.g., Au, Pt) and inorganic semiconductors (e.g., Si).^18,28^ Their chronic applications are hindered by their high electrochemical impedances, low charge injection capacities,^12^ and limited long-term functional stability.^29^ The emergence of new materials such as 2D nanomaterials (e.g., graphene, MXene),^16,30,31^ conductive polymers (e.g., poly(3,4-ethylenedioxythiophene)-polystyrenesulfonate (PEDOT:PSS)),^32−34^ and dielectric metal oxides (e.g., iridium oxide)^5,35^ has opened opportunities for high-performance bioelectronics.^11^ However, these materials are currently limited by their 2D topology, material degradation, or substrate delamination during chronic operation.^5,29^ An alternative approach to enhancing the functional performance of bioelectronics while leveraging the unique benefits of each material class is through the development of hybrid nanomaterials.^1,29^

We recently reported a breakthrough graphene-based hybrid nanomaterial that significantly improves the electrical properties needed for neuronal interfaces. Hierarchical nanowire-templated 3D fuzzy graphene (NT-3DFG) allows the construction of a truly 3D hybrid nanostructure.^36^ NT-3DFG is composed of vertically standing single- to few-layer graphene flakes on isolated Si nanowires (SiNWs).^4,36^ The out-of-plane growth of graphene sheets expands the exposed surface area, improving cell coupling and electrochemical performance.^4,36−38^ Conformal templating, referring to the growth of a 2D film on a 3D surface, of the conductive polymer PEDOT:PSS on individual NT-3DFG nanowires through electropolymerization results in a hybrid nanomaterial, allowing us to leverage the exceptional surface area of NT-3DFG and the volumetric charge storage properties of PEDOT:PSS synergistically to enhance recording and stimulation capabilities (Figure 2b). Our tunable bottom-up growth techniques enable NT-3DFG electrodes to exhibit a range of structural (electrodes ranging from 2 to 200 μm in diameter) and chemical properties (Figure 2c).^4,15,36,39,40^ Precise control over the size and density of vertically aligned graphene flakes and edges enables a scalable electrochemical surface area (ECSA) readily available for either sensing or catalysis applications (Figure 2d).^1,3,4,36,37^

Input bioelectronics facilitates the modulation of cellular electrophysiology and information transduction to the interfaced cells and tissues. Ideal electrical stimulators should exhibit a capacitive response rather than a Faradaic response to avoid the electrolysis of media and the oxidation of metabolites as well as to maintain a stable electrode–electrolyte interface.^5,41^ To avoid the electrolysis of H_2_O in aqueous media, the potential window in cyclic voltammetry (CV) scans should be maintained within the water electrolysis window of an employed electrode.^5,41^ Electrical stimulation is generally achieved through a series of biphasic current pulses with cathodal and anodal phases.^5,41^ The capacitive and Faradaic currents generated at the cell membrane during the cathodal current phase at the stimulating electrode lead to the depolarization of the membrane and result in neuronal activation.^5^ To prevent cellular damage during electrical stimulation, the maximal cathodic potential drop (*E*_*mc*_) and the maximal anodic potential drop (*E*_*ma*_) across the electrode–electrolyte interface is governed by the CIC of the microelectrode,^5^ determined as the amount of charge that can be injected without an *E*_*mc*_ crossing the electrochemical potential window (assessed through voltage transient measurements).^42^ The CIC of NT-3DFG microelectrodes was determined to be up to *ca*. 10-fold greater than that of Pt (Figure 2e). NT-3DFG’s CIC of up to 3.66 ± 1.42 mC cm^–2^ is at least 1 to 2 orders of magnitude greater than those reported for 2D graphene and 3D carbon nanostructures.^43,44^ Although electrodeposited PEDOT:PSS exhibits greater CIC than NT-3DFG at larger electrode diameters, 20 μm NT-3DFG electrodes have comparable CICs. The relatively lower CIC of NT-3DFG may be attributed to the intricate pores between graphene flakes grown on individual SiNWs that are expected to have high charge injection time constants.^5^ The conformal coating of PEDOT:PSS on individual NT-3DFG circumvents this limitation by boosting the CIC to up to *ca*. 30-fold greater than those of Pt microelectrodes (Figure 2e). The CIC of up to 13.21 ± 3.64 mC cm^–2^ of NT-3DFG conjugated with PEDOT:PSS (NT-3DFG+PEDOT:PSS) is *ca*. 10-fold greater than that of the recently reported 3D graphene microelectrodes with close-packed PEDOT:PSS.^45,32^ We observed that the CIC of NT-3DFG and NT-3DFG+PEDOT:PSS increased from 0.81 ± 0.02 to 3.66 ± 1.42 mC cm^–2^ and from 3.31 ± 0.43 to 13.21 ± 3.64 mC cm^–2^ as the electrode diameter decreased from 200 to 20 μm, respectively.^1^ NT-3DFG and hybrid NT-3DFG+PEDOT:PSS are efficient electrode materials for input bioelectronics, enabling potential I/O bioelectrical interfaces.

Electrochemical impedance provides a direct estimate of the recording capabilities of an electrode.^5,41^ Designing bioelectrical interfaces with low impedances is important for enhancing SNR during electrophysiology recording.^19,46^ The electrochemical impedance of NT-3DFG microelectrodes was observed to be more than an order of magnitude lower than that of conventional Pt microelectrodes of similar sizes, with drastic differences apparent in low- to mid-frequency ranges (1–5,000 Hz). We attribute this to the much greater exposed surface area of the NT-3DFG microelectrodes compared to that of the conventional 2D microelectrode.^11,24^ Templating PEDOT:PSS onto NT-3DFG further lowered the electrochemical impedance of the electrodes due to the mixed electronic and ionic conductivities and the volumetric charge storage capacity of PEDOT:PSS (Figure 2f).^5^ The electrical recording capabilities of NT-3DFG microelectrodes were investigated by interfacing human embryonic stem cell-derived cardiomyocytes (hESC-CMs) with 2, 5, and 10 μm NT-3DFG circular electrodes (Figure 2g). These results highlight the importance of using microelectrodes that enable higher spatial resolution and minimize the averaging of signals which cannot be achieved with electrodes similar to or larger than a cell.^37^

The enhanced electrochemical properties of the hybrid nanomaterial can facilitate further miniaturization of bioelectronic interfaces to ultramicroelectrodes for high spatial resolution I/O application. Despite the high realized performances, the organic–inorganic heterostructure of NT-3DFG+PEDOT:PSS highlighted here represents only an initial foray into the potential of hybrid technologies possible with the current expansive material libraries for electrode engineering. The next-generation I/O bioelectronics will exhibit enhanced performance through multimaterial nanoscale engineering.

## Multiscale Optical Interfaces

**Figure 3:**
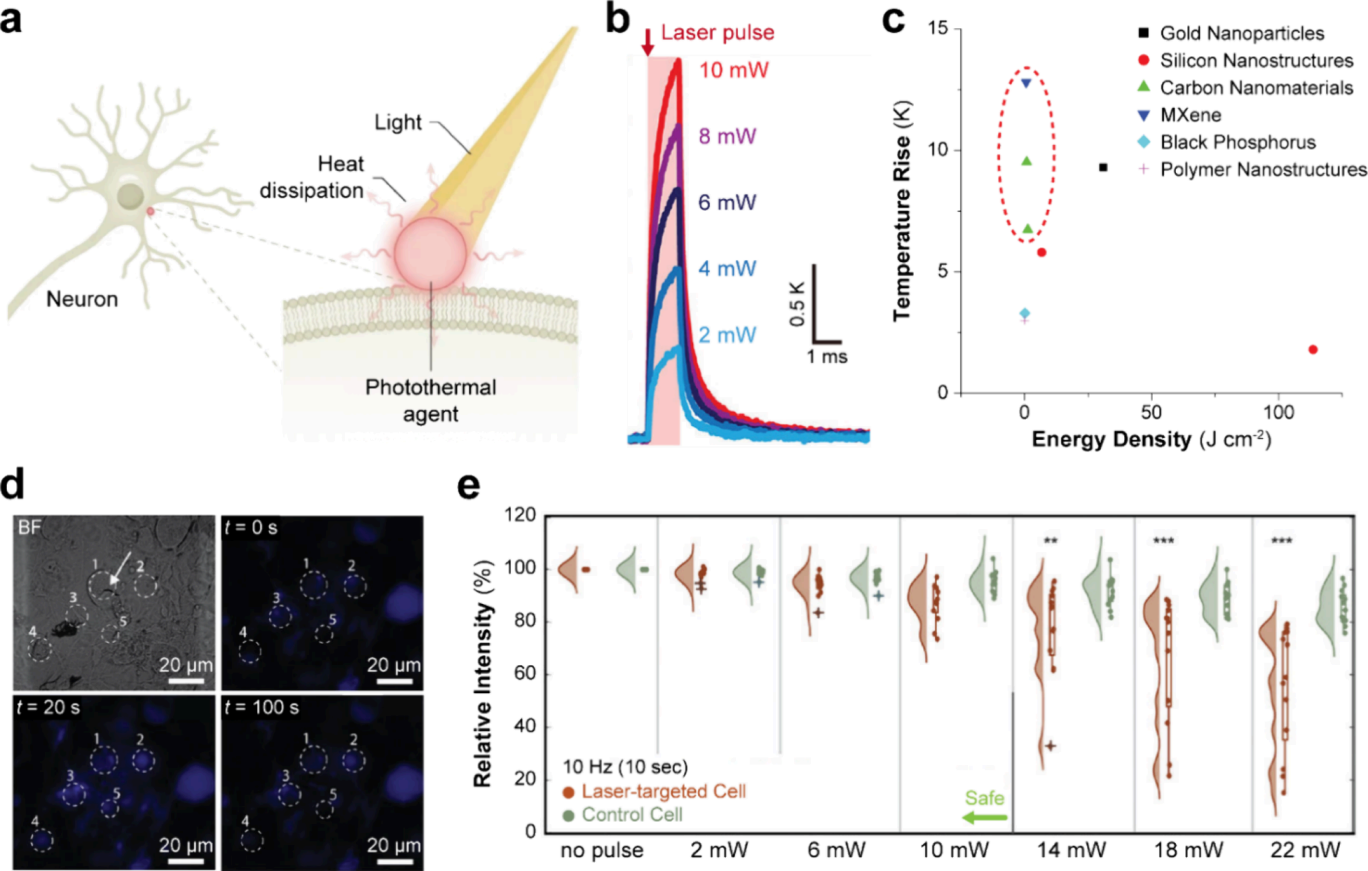
Bidirectional optical neural interfaces enabled by nanomaterials. (a) Schematic illustration of the nanomaterial-enabled photothermal stimulation of neurons. (b) Threshold illumination energy across multiple material classes and wavelengths, with highlighted applications for titanium carbide MXene and NT-3DFG denoted by a dashed circle. (c) Local temperature rise from a MXene thin film measured through a micropipette capillary technique. (d) Ca^2+^ fluorescence imaging of neurons stimulated optically through an application of MXene flakes and (e) the phototoxicity of such stimulation quantified through cell membrane integrity. Reprinted with permission from ref (2), copyright 2021 American Chemical Society; ref (48), copyright 2023 John Wiley and Sons; and ref (53), copyright 2023 Springer Nature.

Despite most human neurons possessing no intrinsic light sensitivity, optical neuronal interfaces have been established as a secondary route for bidirectional communication for more than two decades.^47^ The stimulation of neurons was originally enabled by either high-power laser heating or genetic modification for the expression of light-sensitive ion channels through optogenetics. Optical neuronal recording can be realized through the imaging of fluorescent dyes or endogenously expressed fluorophores that mark intracellular calcium ions (Ca^2+^), membrane potential, or additional cell state indicators (e.g., protein production/accumulation, neurotransmitter concentration).^48^ This mode of recording is well established, with measurement depths provided by two-photon microscopy. Millimeter-scale tissue penetration can be achieved by implantable waveguides for bidirectional optical interfaces both *in vitro* and *in vivo*.^49−51^ However, optical stimulation has faced numerous challenges with direct laser stimulation, inducing cellular damage. Optogenetics has similarly faced limitations in the induction of immune responses in vivo, limited success in gene delivery, and the intrinsic irreversibility of such modifications.^52^ To circumvent these limitations, material-assisted photostimulation, where an absorber is placed within the proximity of a target cell (Figure 3a), has been adapted to convert illumination into an excitatory or inhibitory neural response.

Organic and inorganic nanomaterials have been widely adapted to optical neural modulation for their high photoconversion efficiencies and versatility through either photothermal, photo-Faradaic, or photovoltaic effects.^53^ The photothermal effect in particular is valued for inducing membrane depolarization through optocapacitive coupling of a material to an adjacent cell through light-induced heating^54^ but requires materials with high absorption coefficients ideally over a near-infrared (NIR) biological optical window. 2D titanium carbide MXenes (Ti_2_C_3_T_*x*_) have shown promise for their exceptional surface plasmon resonance absorption in the NIR region, biocompatibility, and broadly tunable optical and electrical behavior.^55^ MXenes exhibit temperature spikes surpassing 10 K with an exceptionally low power threshold of 0.6 J cm^–2^ for a 1 ms pulse. Single MXene flake or thin films were demonstrated to remotely and nongenetically stimulate single neurons (Figure 3b–d).^2^ With such high local temperature fluctuations, concerns have been raised about the potential for such a temperature gradient to damage adjacent cells or produce harmful electrochemical species. To address these concerns, a thorough study was performed by displaying not only biocompatibility during the operation from cell viability but also no significant phototoxicity from reactive oxygen species (ROS) generation, membrane damage, or mitochondrial stress (Figure 3e).^48^ With both the lack of operation-related phototoxicity and reliable, high spatiotemporally resolved neuron photostimulation, MXenes and additional nanocarbons (e.g., NT-3DFG) present a promising route toward translatable stimulation platforms for optical neural interfaces. NT-3DFG, previously applied as an electrical stimulation and recording technology, demonstrates nearly as efficient optical neural stimulation through the photothermal effect as MXenes with complete biological stability,^15^ unlike the transient nature of Ti_3_C_2_T_*x*_.^56^ MXenes and other photothermal agents (e.g., Au nanoparticles) exhibit a limited absorption spectrum (e.g., the near-infrared surface plasmon resonance window in Ti_3_C_2_T_*x*_),^2^ whereas NT-3DFG acts as a broadband absorber with enhanced light-trapping for more versatile application. Both 2D cultures of dorsal root ganglion (DRG) neurons and 3D cortical spheroids were cultured and interfaced with NT-3DFG. Illumination wavelengths from 405 to 635 nm were applied to stimulate neurons with powers smaller than 100 nJ per pulse for both the 2D and 3D cases. The cellular response was gauged through patch-clamp recordings or transient intracellular calcium concentrations through fluorescence imaging. The generated action potentials closely correlated with the electrically stimulated references, with among the highest measurable temperature transients achieved to date. This demonstrates the potential for these nongenetic, nanomaterial-based optical stimulators in three-dimensional cell bodies, which is a critical requirement for the largely unexplored translation of these technologies to animal models or complex 3D organoid systems.

Toward establishing the next generation of optical platforms, the key interest is developing materials that transduce light at high efficiencies. The photothermal approach has seen great success in this area with among the lowest threshold illumination intensities and demonstrated biocompatibility but is capped in application from the requirement of tight cell–material interfaces required to maximize the optocapacitive effect.^2,15,38^ For this reason, approaches where interface coupling is maximized are becoming more prevalent, especially those that utilize the photovoltaic effect to directly generate voltages.^57^ Higher-complexity material considerations in constructing functioning photovoltaics at the neuron scale have thus far lead to relatively lower photoconversion efficiencies but in conjunction with enhanced capacitive coupling can lead to a significantly decreased overall power threshold not solely limited to *in vitro* experimentation.^57−59^ As the newly developed materials and optical interfaces grow more powerful, the potential of entirely remote bidirectional communication with neurons *in vivo* becomes increasingly achievable and could open a new avenue for translatable neural interface technologies.

## Comprehensive Understanding and Modulation of Neuronal Microenvironments

**Figure 4:**
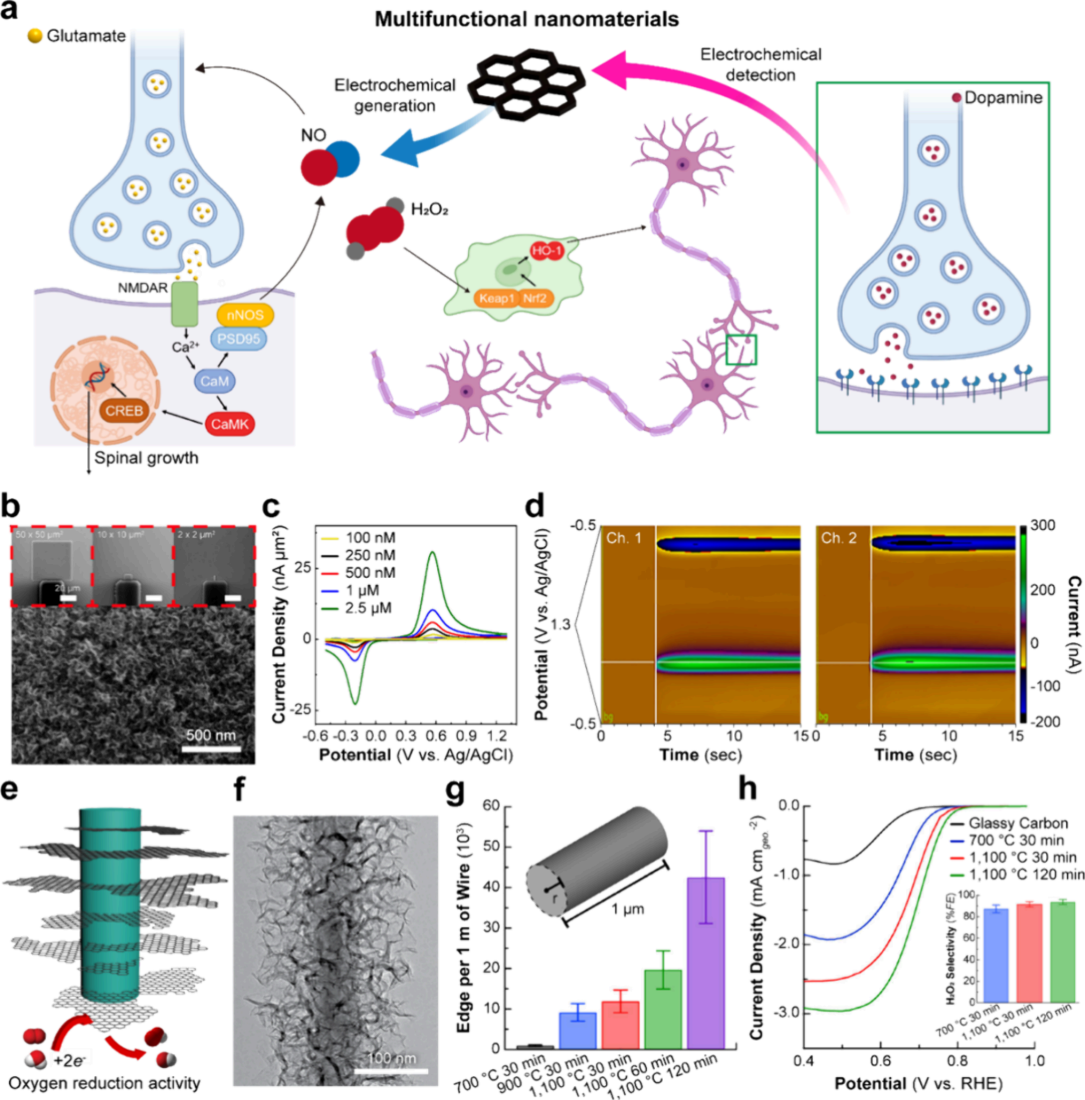
Electrochemical systems for detecting and generating small molecular biomarkers. (a) Schematic illustration of multifunctional nanomaterial-enabled detection and modulation of the expression of molecular biomarkers and the mechanism of such biomarkers in neurological cascades. (b) Microfabricated 3D fuzzy graphene (3DFG) microelectrode arrays (MEAs). (c) Dopamine detection capability via fast scan cyclic voltammetry and (d) a demonstration of multisite dopamine sensing using miniaturized 3DFG MEAs (2 × 2 μm^2^). (e) Schematic illustration of the oxygen reduction reaction (ORR) in NT-3DFG. (f) Representative transmission electron microscopy (TEM) image of NT-3DFG. (g) Engineered edge density by varied synthesis conditions. (h) Linear sweep voltammetry (LSV) scan in the cathodic direction to investigate electrocatalytic activities in ORR. Inset: selectivity toward hydrogen peroxide generation via 2-electron pathway. Panels (b–h) reprinted with permission from ref (3), copyright 2021 Elsevier, and ref (4), copyright 2021 American Chemical Society.

The superior electrical performances of nanoscopic materials have contributed to the advancement of conventional neural interfaces. Recent neurotechnology breakthroughs have demonstrated nanomaterial uses beyond the direct modulation (or recording) of neural membrane potential.^17^ However, both short- and long-range neural communication are dominated by neurotransmitters and small molecules.^60,61^ Thus, understanding cellular chemical environments can provide comprehensive insight into (patho)physiology, while moving further into manipulating this environment on the microscale would provide unprecedented control over cellular behavior. Electrochemical tunability of nanomaterials enables bioelectronics for electrocatalytic generation and highly selective detection of signaling molecules that initiate and govern physiological cascades. Such bioelectronics could detect anomalous expression of neurochemicals that represent neurological disorders and neurodegenerative diseases. Additionally, these small molecular factors can be produced by catalytic reactions through multifunctional nanomaterials. By combining the capability of monitoring and producing certain signaling molecules, we can construct “smart” multifunctional bioelectronics which are operated in a closed-loop manner for simultaneous diagnosis and therapeutic treatment (Figure 4a). To detect and monitor the levels of small molecular factors involving neurological transmission (e.g., nitric oxide-mediated glutamate transmission), there have been extensive efforts including through optical and electrical methods. Electrochemical detection has been reported as a promising approach due to its superior sensitivity, rapid detection, and relatively low cost of the required equipment for sensing.

Dopamine (DA) has been regarded as one of the most important analytes in neural systems.^62−64^ Since its abnormal expression is highly relevant to diverse neurological disorder (e.g., schizophrenia and Parkinson’s disease),^62,63^ monitoring the concentration of DA with high spatial and temporal resolution is crucial to understanding and diagnosing such diseases as well as tracking their progress and developing treatments. While carbon fiber electrodes (CFEs) coupled with fast scan cyclic voltammetry (FSCV) have been the gold standard for *in vivo* DA sensing,^60,65^ FSCV measurements using CFEs face several drawbacks. A lack of multisite sensing capabilities and suboptimal selectivity toward DA detection calls for the development of highly selective biosensing arrays with high electrode density, which will realize rapid and accurate detection in minimal dimensions with quick responses. Recently, a family of nanocarbon materials including CNTs,^65^ nanodiamonds,^66^ and graphene oxide^67^ has been investigated for DA detection, coated on CFE-based sensors to improve sensitivity, selectivity, and resistance to fouling.

Graphene is a promising nanocarbon candidate due to its fabrication versatility via either top down or bottom up. Graphene’s basal plane is electrochemically inactive owing to robust *sp*^2^ hybridization,^67^ thus only the defects or edges can play a role as electrochemically active sites. For this reason, researchers have made efforts to engineer structural defects and controlled edge density of synthesized graphene. 3DFG offers edge-abundant graphene nanostructures, which can construct micro- and nanofabricated microelectrode arrays (Figure 4b). The defect-rich nanocarbon material results in improved DA detection performance (Figure 4c). 3DFG MEAs were shown to achieve a limit of detection (LOD) of 364.44 ± 8.65 pM and a sensitivity of 2,120 ± 50 nA μM^–1^ with selectivity from potential interferences such as uric acid (UA), ascorbic acid (AA), dihydroxyphenylacetic acid (DOPAC), epinephrine (EP), and 5-hydroxyindole-3-acedic acid (5-HIAA). Compared to CFE-based DA detection using FSCV, the microfabricated 3DFG MEAs outperform CFEs in LOD (218 nM) and sensitivity (49 ± 0.5 nA μM^–1^).^64^ More importantly, exceptionally small dimensions of MEAs realized by bottom-up microfabrication (down to 2 × 2 μm^2^) demonstrated the feasibility of a high-density, multichannel DA biosensing platform utilizing FSCV (Figure 4d). The 3DFG-based multichannel DA biosensors enable highly spatially resolved mapping of the DA distribution. With a spacing down to 25 μm, each electrode in the MEA can detect DA independently without cross-talk across adjacent electrodes.

A new neurotechnological deployment of nanomaterials is local, highly controlled chemical cellular modulation. Recently, it has been reported that nanomaterial-enabled electrocatalysis is capable of generating small signaling molecules that affect biological processes,^68^ including carbon monoxide (CO), nitric oxide (NO), and hydrogen peroxide (H_2_O_2_) which are ubiquitous in various neurological cascades such as stimuli of dopaminergic neuron maturation, spine growth, and neurotransmission.^61,69,70^ A certain concentration of such neurochemicals triggers a specific neurological cascade and maintains it for homeostasis. Meanwhile, an abnormal expression of these small molecular biomarkers can deviate from the feedback cycle from homeostatic neurological processes, resulting in various types of diseases eventually. Thus, the production of signaling molecules and neurochemicals (e.g., CO, NO, O_2_, H_2_O_2_, and reactive oxygen species (ROS)) allows the modulation of the chemical composition in the intra/intercellular environment, sequentially enabling the manipulation of physiological feedback loops by upregulating a certain corresponding molecule.

Electrocatalysis can be used for the direct delivery of neurochemicals. Relatively small molecular factors (e.g., CO, NO, O_2_, and H_2_O_2_) can be readily generated through electrochemical reactions in physiological fluids. While these chemicals are produced by enzyme-mediated reactions in native environments, a proper electrocatalyst can generate the small molecules upon appropriate potential application. The Anikeeva group and colleagues demonstrated the *in situ* electrocatalytic reduction of nitrite ions (NO_2_^–^) using iron sulfide nanocrystals.^71^ The electrochemical reaction leads to a local elevation of NO levels, which induced NO-mediated neuronal excitation in the targeted brain region and its excitatory projections.

Moreover, hydrogen peroxide is an important biomarker in diverse physiological cascade reactions such as apoptosis,^72^ immune responses,^73^ and beyond. In the neurological system, the concentration of hydrogen peroxide is related to the nuclear factor erythroid 2-ralted factor (Nrf2) signaling pathway.^69^ Recognized as an intracellular regulator of neuronal growth,^74^ Nrf2 triggers the expression of antioxidant proteases including heme oxygenase-1 (HO-1) and NADPH quinone oxidoreductase-1 (NQO1) (Figure 4a). The high level of expression of such enzymes is closely related to neurodegenerative diseases,^75−78^ thus H_2_O_2_-induced Nrf2 activation is pivotal to understanding the progression of Parkinson’s disease as well as treatment.

While hydrogen peroxide is formed in the body by enzyme-mediated reactions, it can be obtained from water via the oxygen reduction reaction (ORR). The yield of H_2_O_2_ is determined by the electrochemical pathway of ORR. While the 4-electron pathway results in the formation of water (acidic condition) or hydroxide anions (OH^–^, neutral/alkaline condition), H_2_O_2_ and hydrogen peroxide anions (HO_2_^–^) are generated via the 2-electron pathway under acidic and neutral/alkaline conditions, respectively. For the effective production of H_2_O_2_, it is pivotal to select proper materials which present the two-electron oxygen reduction mechanism as the dominant pathway of ORR. In recent decades, carbon-based catalysts have captured many researchers’ attention due to their cost-effectiveness and exceptional stability. While noble metal alloys (e.g., Pd–Au and Pt–Hg)^79,80^ have been regarded as the benchmark, given their low overpotential and high selectivity toward H_2_O_2_ production, their scarcity and cost limit their ultimate scalability. Current breakthrough engineering of nanocarbon materials enables the efficient generation of H_2_O_2_. A great number of defects and edges in 3DFG can provide a plethora of active sites for electrocatalysis. We reported that NT-3DFG showed remarkably selective hydrogen peroxide generation via 2-electron ORR (Figure 4e–h).^4^ Notably, the edge density can be engineered under different synthesis conditions (Figure 4g). As described earlier, edge density is a key aspect of graphene-based electrochemical materials. By manipulating the density of edges, NT-3DFG can be tuned to generate the proper level of H_2_O_2_ in physiological setups. Exhibiting multifunctionality in electrophysiology, electrochemical sensing, and electrocatalysis, NT-3DFG can build up multimodal bioelectronics for the comprehensive sensing and modulation of neuronal systems.

*In vitro* and *in vivo* chemical modulating via electrocatalysis is limited by efficient catalysts in physiological settings. For example, effective electrocatalysts for water electrocatalytic reactions such as the hydrogen evolution reaction and oxygen evolution/reduction reaction (OER/ORR) have been extensively researched in energy applications. Unlike the pH-neutral biological environment, energy-based electrocatalysts are being employed under extreme conditions (e.g., strong acidic/alkaline and high temperature). We recently demonstrated that nanomaterial-based electrocatalytic arrays can effectively modulate the cellular chemical environment, particularly for oxygen at neutral pH. The electrocatalytic system generated oxygen microgradients within implanted cells in an animal model by utilizing a sputtered iridium oxide film (SIROF) to catalyze OER.^81^ Nanostructured SIROF has been widely investigated in electrophysiological studies owing to its stability, biocompatibility, reversible Faradaic charge transfer, and superior charge injection capacity derived from nanoscopic morphologies.^35^ Since larger ECSA contributes to the greater number of active sites for electrochemistry, electrocatalytic water oxidation can be more efficiently conducted even in kinetically unfavored neutral pH. The devised system demonstrated highly selective oxygen production in complex media (1× phosphate buffer saline, pH 7.4) without expected byproducts during electrochemical water splitting. Oxygen produced by the electrocatalytic reaction contributed to improved cell viability in high-density (60K cells mm^–3^) alginate cell capsules under hypoxic incubation (1% O_2_) as well as peptide-producing capabilities both *in vitro* and *in vivo*.

Maintenance of implantable cell therapies with high cell density by generating oxygen highlights the potential of electrocatalytic approaches in neurotechnology. Transplantable cell therapies can be applied as drug delivery systems for neurological disorders and neurodegenerative diseases. For instance, it has been reported that the accumulation of amyloid plaque in the brain can be ameliorated by delivering amyloid beta (1–17) dimers to suppress the progress of Alzheimer’s disease.^82^ Employing engineered cells to produce the peptide, cell therapeutics could potentially be utilized in the treatment of neurological disorders in such a fashion.

Integrating electrocatalytic platforms and sensing systems, therefore, is highly crucial to accomplish closed-loop bioelectronics for neurotransmission and other physiological cascades in the nervous system. Although only a few studies have been reported, electrocatalytic manipulation of chemical compositions in the neuronal system can open enormous opportunities to control and modulate neurological behaviors. Coupled with neurotransmitter and neurochemical detection, the deployment and integration of electrocatalysts in neural-interfacing bioelectronics will provide more comprehensive tools to investigate the chemical communication of cells and to construct better approaches for the treatment of neurological disorders and degenerative diseases in a closed-loop manner.

## Summary and Outlook

As the spatiotemporal resolution, recording quality, invasiveness, and stability for neural interfaces continue to advance, the limiting bottleneck is shifting away from platform-side engineering toward that of their materials. Electrical platforms demonstrating performance improvement through nanomaterial heterostructure decoration, optical modulation bypassing the need for the genetic modification of neurons with among the lowest light power thresholds, and control of the electrochemical environment surrounding neurons through selective catalysis and sensing have been developed, with some utilizing the same material class across broad applications. Through the existing vast libraries of materials for translation across semiconductors, batteries, photovoltaics, catalysis, and beyond, the device performance improvements of scale seen in these works across all modalities are just beginning. There is a large uptick in public attention toward the development of brain–computer interface technologies and a yet unseen prominence of commercial-scale application; therefore, the next decade poses an essential opportunity in locating and optimizing multiple modalities for bidirectional communication with neural circuits. With this centering goal and the continued translation of cutting-edge materials science to bioelectronics, the next generation of neural interfaces will be defined by their structure not at the millimeter scale but rather by the nanometer.
